# Improvement in pH Sensitivity of Low-Temperature Polycrystalline-Silicon Thin-Film Transistor Sensors Using H_2_ Sintering

**DOI:** 10.3390/s140303825

**Published:** 2014-02-25

**Authors:** Li-Chen Yen, Ming-Tsyr Tang, Fang-Yu Chang, Tung-Ming Pan, Tien-Sheng Chao, Chiang-Hsuan Lee

**Affiliations:** 1 Department of Electrophysics, National Chiao Tung University, Hsinchu 30010, Taiwan; E-Mails: karytan@hotmail.com (L.-C.Y.); vicky1468@hotmail.com (M.-T.T.); lovegood.am96@gmail.com (F.-Y.C.); tschao@mail.nctu.edu.tw (T.-S.C.); 2 Department of Electronics Engineering, Chang Gung University, Taoyuan 333, Taiwan; 3 Department of Nuclear Medicine, Chi Mei Medical Center, Tainan 710, Taiwan

**Keywords:** low-temperature polycrystalline-silicon (poly-Si), thin-film transistors (TFTs), H_2_ sintering, pH sensitivity

## Abstract

In this article, we report an improvement in the pH sensitivity of low-temperature polycrystalline-silicon (poly-Si) thin-film transistor (TFT) sensors using an H_2_ sintering process. The low-temperature polycrystalline-silicon (LTPS) TFT sensor with H_2_ sintering exhibited a high sensitivity than that without H_2_ sintering. This result may be due to the resulting increase in the number of Si–OH_2_^+^ and Si–O^−^ bonds due to the incorporation of H in the gate oxide to reduce the dangling silicon bonds and hence create the surface active sites and the resulting increase in the number of chemical reactions at these surface active sites. Moreover, the LTPS TFT sensor device not only offers low cost and a simple fabrication processes, but the technique also can be extended to integrate the sensor into other systems.

## 1. Introduction

pH is one of the most common laboratory measurements because so many chemical and biological processes rely on pH. The body fluids of living organisms usually survive within a narrow pH range. If the pH value of the human blood changes by as little as 0.03 pH units or less, many body functions will be greatly impaired [1]. Measuring pH is essential not only in finding the chemical characteristics of a substance but also as the first step toward managing chemical reactions. Consequently, exact pH measurement is also carried out in nearly all industries. The ion-sensitive field-effect transistor (ISFET) was first proposed for pH sensing by Bergveld in 1970 [2]. In recent years, it has received much attention for various purposes in biomedical, medicine, and chemical applications [3–4]. In ISFET devices, the metal gate electrode of metal-oxide-semiconductor field-effect transistors (MOSFETs) is replaced by the series combination of the reference electrode, electrolyte and chemically sensitive membrane. ISFET sensors have demonstrated very fast response, high sensitivity, and high potential for integrating complementary metal-oxide-semiconductor (CMOS) technology [5]. The gate oxide of an ISFET sensor is in direct contact with the electrolyte solution. The high surface buffer capacity in ISFET device causes an almost constant proton density at the surface and is independent of the electrolyte's pH [6]. To equalize the free energy difference for protons at the interface and in the bulk of the solution, the pH-dependent potential drop across the double layer arises, leading to the modulation of the channel conductance.

Low-temperature polycrystalline-silicon (LTPS) thin-film transistors (TFTs) have been developed to function as transducers for ultrasensitive and label-free detection of biomolecules, such as proteins, DNA, glucose, viruses, *etc.*, due to their excellent electrical characteristics in aqueous solution [7–11]. Furthermore, they are easily mass-produced a simple, low-cost, and low-temperature processes. However, the sensitivity is seriously affected by the presence of trapped states at the grain boundaries of the poly-Si. To address this issue, hydrogen treatment [12] and fluorine-ion implantation [13] on poly-Si films have been employed to effectively improve the device performance by reducing the trap state densities. In this paper, we report an improvement in the pH sensitivity of LTPS TFT sensors using H_2_ sintering.

## 2. Experimental Section

**Figure 1.**
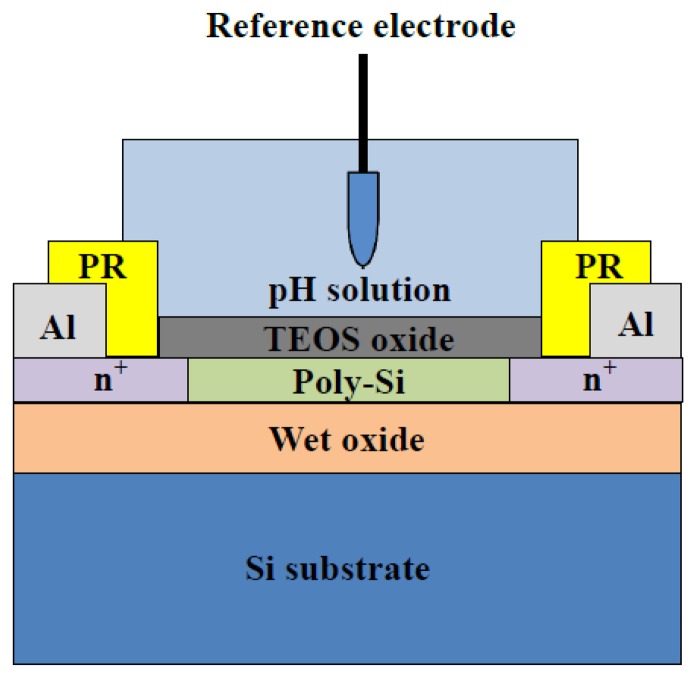
Schematic cross-sectional view of the LTPS TFT sensor devices.

Figure 1 shows the structure of LTPS TFT sensor device. N-channel LTPS TFTs (*W*/*L* = 10 μm/10 μm) were fabricated on 6 inch p-type silicon substrates (100) with a 500 nm thermal oxide layer. First, a 100 nm undoped amorphous-Si (α-Si) layer was deposited on thermal oxide using a low-pressure chemical vapor deposition system (LPCVD) at 550 °C. Next, the α-Si layer was crystallized by solid-phase crystallization (SPC) at 600 °C for 24 h in N_2_ ambient. The source and drain (*S*/*D*) regions were formed by implanting arsenic atoms with an implant energy of 70 keV and a dose of 5 × 10^15^ cm^−2^. After the arsenic implantation, the layer was activated through a furnace at 600 °C for 24 h. The device active region was formed by patterning and dry etching. Then, an 8 nm tetraethoxysilane (TEOS) oxide film was deposited through LPCVD at 650 °C. After the pattering of *S*/*D* contact holes, a 300 nm Al-Si-Cu metal electrode was deposited by physical vapor deposition at room temperature and patterned as the *S*/*D* contact pads. Samples were then sintered at 300 °C in H_2_ gas for 30 min. Finally, the *S*/*D* metal pads were covered with the photo resistor to prevent leakage current from *S*/*D* in aqueous solution.

The pH sensing performance of the LTPS TFT sensors was evaluated using standard buffer solutions (Alfa Aesar Inc., Ward Hill, MA, USA) having values of pH ranging from 2 to 12. To saturate the ionic properties of the TEOS sensing membranes, the LTPS TFT sensors were immersed in reversed osmosis (RO) water for 12 h prior to measurement. The I_DS_-V_GS_ curves for the sensors in the standard buffer solutions at different values of pH were then measured through an Ag/AgCl reference electrode using a Keithley 4200 semiconductor characterization system (Keithley Instruments, Inc., Cleveland, OH, USA) to maintain the equilibrium of the electrochemical system. All of the experimental setups were kept in the dark to avoid any interference caused by ambient light. The threshold voltage is defined as the gate voltage at which the drain-current reaches 1 nA × *W*/*L* at *V*_DS_ = 1 V. Photoluminescence (PL) spectra were measured using a 0.5 m monochromater and detected with a liquid-nitrogen-cooled extended InGaAs photodiode. The excitation source was the 325 nm line of a He-Cd laser. All the measurements were made at room temperature.

## 3. Results and Discussion

**Figure 2.**
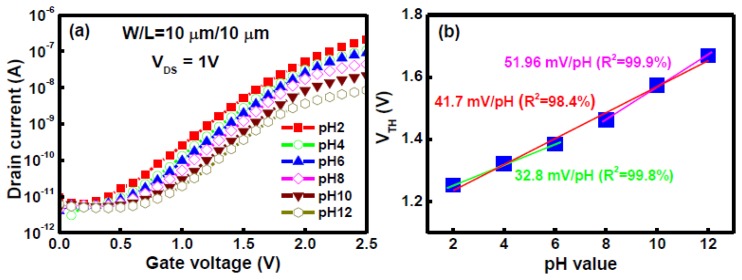
(**a**) Transfer characteristics of a LTPS TFT sensor with H_2_ sintering for pH values from 2 to 12. (**b**) Threshold voltage as a function of pH for a LTPS TFT sensor measured at room temperature.

Figure 2a shows the transfer characteristics I_DS_-V_GS_ of the LTPS TFT sensor device. The LTPS TFT device and reference electrode (RE) were immersed in the pH 2, 4, 6, 8, 10, and 12 buffer solutions for 1 min. The I_DS_-V_GS_ curves were then measured with a positive bias applied to the gate. In pH sensing, the change in pH of a solution causes a shift of the threshold voltage in the I_DS_-V_GS_ curve, mainly due to the ionization of surface OH groups by either H^+^ or OH^−^. Furthermore, the I_DS_-V_GS_ curves were shifted parallel with decreasing the concentration of hydrogen ion under positive bias. This phenomenon can be explained by the surface site-binding model [14]. The threshold voltages of LTPS TFT sensor immersed in various pH solutions were extracted from the I_DS_-V_GS_ curve to determine its pH sensitivity. Figure 2b demonstrates that a sensitivity of 41.7 mV/pH and alinearity of 98.4% were calculated by linear fitting from pH 2 to pH 12. We also found that there are two slope sections: one is a low sensitivity zone of 32.8 mV/pH (*R*^2^ = 99.8%) at a pH ranging from 2 to 6 and another is a high sensitivity region of 51.96 mV/pH (*R*^2^ = 99.9%) at a pH ranging from 8 to 12, as shown in Figure 2b. This behavior may be attributed to the small β value. The chemical reactions at the surface of a gate oxide were discussed based on the site-binding model when it is in direct contact with the electrolyte solution. We assume that the oxide surface contains sites in three possible forms: Si-OH, Si-O^−^, and Si-OH_2_^+^. The surface reactions are described as follows:

(1) $$\begin{array}{c} \text{Si}-\text{OH}\overset{{\text{K}}_{-}}{\Leftrightarrow }\text{Si}-{\text{O}}^{-}+{\text{H}}_{\text{S}}^{+}\\ \text{Si}-{\text{OH}+\text{H}}_{\text{S}}^{+}\overset{{\text{K}}_{+}}{\Leftrightarrow }\text{Si}-{\text{OH}}_{2}^{+} \end{array}$$

where H_S_^+^ is the surface concentration of H^+^ ions, K_−_ and K_+_ are the acidic and basic equilibrium constant, respectively. According to the Gouy-Chapman-Stern theory, the *ϕ_0_* can be obtained from [15]:

(2) $$2.303(pH_{\text{pzc}}-pH)=\frac{q\phi_{.0}}{kT}+\sin h^{-1}(\frac{q\phi_{.0}}{kT}\frac{1}{\beta })$$

where pH_pzc_ is called the pH at the point of zero charge, *ϕ_0_* is the potential drop in the electrolyte at the insulator-electrolyte interface, k is the Boltzmann constant, *T* is the measuring temperature, and β is a parameter, which reflects the chemical sensitivity of the gate oxide and is dependent on the density of surface hydroxyl groups. It is given by:

(3) $$\beta =\frac{2q^{2}N_{s}\sqrt{K_{+}/K_{-}}}{\text{k}TC_{DL}}$$

where *N_s_* is the surface site density and *C_DL_* is the double layer capacitance [9]. If the β value is large enough, Equation (2) becomes:

(4) $$\phi_{0}=-2.303\frac{\text{k}T}{q}\frac{\beta }{\beta +1}({\text{pH}}_{\text{pzc}}-\text{pH})$$

From Equation (3) it can be observed that the response of the sensor depends on β: the higher *N_s_* causing larger β, and thus leading to higher pH sensitivity and the more linear the response. However, the poor behavior of linearity may be due to the small β value and low surface site density.

**Figure 3.**
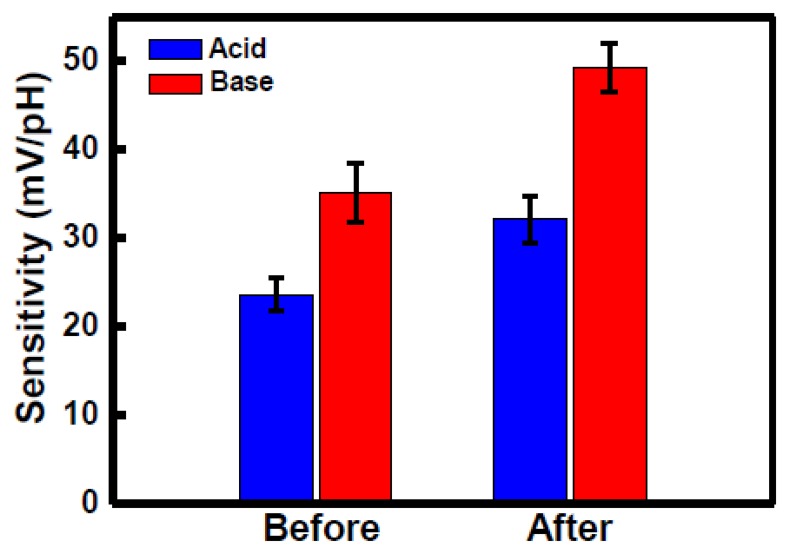
pH sensitivity of the LTPS TFT sensors before and after H_2_ treatment for acidic and basic sides.

**Figure 4.**
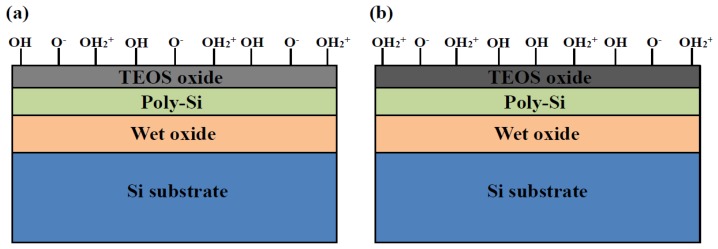
Schematic of the surface site behavior for LTPS TFT sensor (**a**) before and (**b**) after H_2_ treatment.

To reduce the silicon dangling bonds, H_2_ sintering treatment can be used to improve the pH sensitivity of a LTPS TFT sensor device. Figure 3 shows the pH sensitivity of such a LTPS TFT device before and after H_2_ sintering for acidic and basic sides. The TFT sensor with H_2_ sintering exhibited a higher pH sensitivity than that without H_2_ sintering. The hydrogen passivation of a silicon bond may transform the SiO_2_, passivating a more removed silicon bond or linking up with oxygen or even forming H_2_ [16]. The hydrogen can then diffuse through the TEOS oxide film during an H_2_ sintering process in order to reach the oxide–poly-Si interface and passivate the dangling bonds. To balance the surface reactions when the device is immersed in a solution, the hydrogen may escape from the interface to the oxide surface and thus increase the density of surface sites (–OH and –OH_2_^+^), producing a larger β value. Figure 4a illustrates a representation of the site-binding model, where the gate oxide surface contains a large amount of unsatisfied bonds. In the absence of specific adsorption, the only ions capable of making bonds with these sites are the hydrogen and hydroxyl ions. At thermal equilibrium and under no net reaction conditions, Equation (1) is dynamically balanced in the membrane. After H_2_ sintering treatment, the numbers of Si–O^−^ and Si–OH_2_^+^ bonds rise and increase the chemical reactions at surface active sites, as shown in Figure 4b, resulting in a higher pH sensitivity.

**Figure 5.**
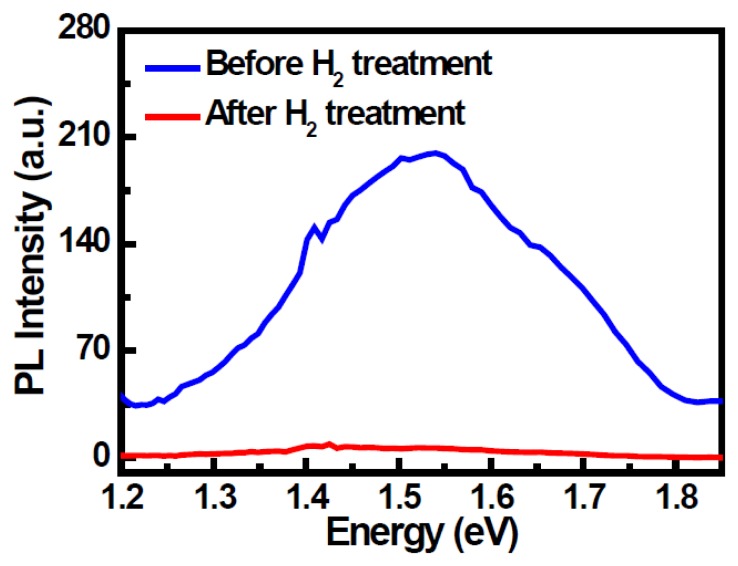
PL spectra of the TEOS oxide film with and without H_2_ sintering treatment.

Photoluminescence (PL) spectroscopy was performed to study the defects of the TEOS film. Figure 5 shows the PL spectra on the gate oxide film with and without H_2_ sintering treatment. The LTPS TFT device with H_2_ sintering exhibited a lower PL peak density compared to that without H_2_ sintering. The incorporation of H in the gate oxide can reduce the silicon dangling bonds at the oxide–poly-Si interface, and thus create surface active sites when the device is immersed in the electrolyte solution.

**Figure 6.**
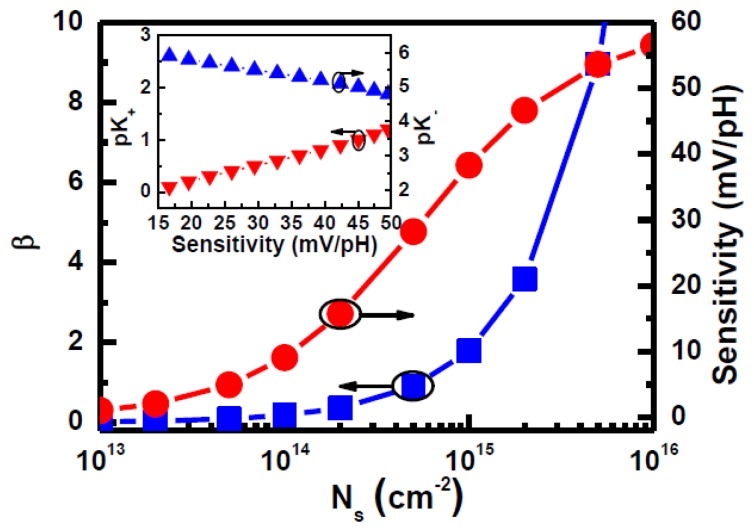
β and sensitivity characteristics of pH sensor simulated at different values of site density. In the inset: the pK_+_ and pK_−_ as a function of pH sensitivity.

We suspect that the pH sensitivity increases because the LTPS TFT sensor has a higher surface site density and/or a chemical changes such as K_+_ and K_−_ after H_2_ sintering treatment. On the other hand, we can obtain a relationship curve of the *ϕ_0_* (pH sensitivity), β, and *N_S_* parameters from Equations (2) and (3), as shown in Figure 6. We assume that the C_DL_ and pH_pzc_ are 2 × 10^−5^ F/cm^−2^ and 3 [11], respectively. The pH sensitivity and β value clearly increase with the increasing *N_S_* value. The pH sensitivity is close to an ideal value (59.54 mV/pH) when the *N_S_* is more than 10^16^ cm^−2^. It was found that the pK_+_ and pK_−_ values can have a significant impact on sensitivity, as shown in the inset of Figure 6. Therefore, the pH sensitivity was improved due to not only to changes in the acidic and basic equilibrium constants but also because the surface site density increased.

## 4. Conclusions

In the paper, we successfully developed the fabrication of LTPS TFT sensors after H_2_ sintering treatment for pH sensing applications. The LTPS TFT sensor with an H_2_ sintering process has better sensing properties than that without an H_2_ sintering process. This outcome may be attributed to the increase in the number of of Si–OH_2_^+^ and Si–O^−^ bonds thus enhancing the chemical reactions at surface active sites. In addition, we have explored the critical parameters to be used for the optimization of the pH sensing ability, such as acidic equilibrium constant, basic equilibrium constant, and surface site density. This device holds great promise for system-on-chip, nonvolatile memory processes, and biosensors applications.
